# A Decade Later, How Much of Rwanda's Musculoskeletal Impairment Is Caused by the War in 1994 and by Related Violence?

**DOI:** 10.1371/journal.pone.0007720

**Published:** 2009-11-05

**Authors:** Hannah Kuper, Oluwarantimi Atijosan, Dorothea Rischewski, Victoria Simms, Chris Lavy

**Affiliations:** 1 Department of Epidemiology and Population Health, London School of Hygiene and Tropical Medicine, London, United Kingdom; 2 Heatherwood and Wexham Park Hospitals National Health Service Foundation Trust, Slough, Berkshire, United Kingdom; 3 Deutsche Gesellschaft für Technische Zusammenarbeit (GTZ) GmbH, Eschborn, Germany; 4 Department of Palliative Care, Policy and Rehabilitation, King's College London, London, United Kingdom; 5 Nuffield Department of Orthopaedic Surgery, University of Oxford, Oxford, United Kingdom; University of Cape Town, South Africa

## Abstract

**Background:**

In 1994 there was a horrific genocide in Rwanda following years of tension, resulting in the murder of at least 800,000 people. Although many people were injured in addition to those killed, no attempt has been made to assess the lasting burden of physical injuries related to these events. The aim of this study was to estimate the current burden of musculoskeletal impairment (MSI) attributable to the 1994 war and related violence.

**Methodology/Principal Findings:**

A national cross-sectional survey of MSI was conducted in Rwanda. 105 clusters of 80 people were selected through probability proportionate to size sampling. Households within clusters were selected through compact segment sampling. Enumerated people answered a seven-question screening test to assess whether they might have an MSI. Those who were classed as potential cases in the screening test were examined and interviewed by a physiotherapist, using a standard protocol that recorded the site, nature, cause, and severity of the MSI. People with MSI due to trauma were asked whether this trauma occurred during the 1990–1994 war or during the episodes that preceded or followed this war. Out of 8,368 people enumerated, 6,757 were available for screening and examination (80.8%). 352 people were diagnosed with an MSI (prevalence = 5.2%, 95% CI = 4.5–5.9%). 106 cases of MSI (30.6%) were classified as resulting from trauma, based on self-report and the physiotherapist's assessment. Of these, 14 people (13.2%) reported that their trauma-related MSI occurred during the 1990–1994 war, and a further 7 (6.6%) that their trauma-related MSI occurred during the violent episodes that preceded and followed the war, giving an overall prevalence of trauma-related MSI related to the 1990–1994 war of 0.3% (95% CI = 0.2–0.4%).

**Conclusions/Significance:**

A decade on, the overall prevalence of MSI was relatively high in Rwanda but few cases appeared to be the result of the 1994 war or related violence.

## Introduction

In 1994 there was a horrific genocide in Rwanda, following years of tension between majority and minority ethnic groups. This resulted in the murder of at least 800,000 Rwandese, many through machete attacks, before killing was abated 100 days after it started in July 1994. There are frequent reports of other appalling acts of violence, including amputation and sexual attacks. The long-term consequences of the violence in terms of burden of disability are not well investigated.[1]

A national survey of musculoskeletal impairment (MSI) was undertaken as a joint venture between the Rwandese Ministry of Health and non-governmental organisations in order to plan and monitor health care and rehabilitation services. [2], [3] The survey demonstrated a large burden of MSI in Rwanda (prevalence = 5.2% 95% CI 4.5–5.9%), and this was mostly untreated.

Our objective for these analyses is to estimate the current burden of MSI attributable to the genocide and related violence within the context of this survey. This will allow us to assess the long-term impact of the genocide a decade after the event, beyond the devastating death toll and to estimate the number of people needing services in Rwanda because of their war-related MSI. It will also allow us to consider the proportion of MSI in Rwanda that is attributable to the genocide to facilitate making generalisations about MSI to other African settings.

## Methods

### Definition of MSI

A national survey of MSI was conducted in November-December 2005. The definition of MSI took as its starting point the standard WHO International Classification of Function (ICF) definition of impairment as “a loss or abnormality in body structure or physiological function”. This definition was made specific to the musculoskeletal system including chronic pain, since the mandate for the survey from the Rwandese Ministry of Health was to assess the prevalence of MSI in Rwanda. [4] The definition used in this survey was: *“a lack of normal structure or function, or an increase in pain or discomfort in the integument, muscles, bone or joints of the body of an individual, that has lasted at least one month and which limits function of the musculoskeletal system.”* [2]

### Sample selection

A nationally representative sample of the population was selected. The updated 2002 national census, which included lists of all the enumeration areas, was used as the sampling frame. [3] 105 nationally representative clusters of 80 people (all ages) were selected through probability-proportionate to size sampling from the sampling frame.

Households within clusters were selected through compact segment sampling.[5] Maps of each selected enumeration area were obtained from the census bureau. The enumeration area was visited two to three days before the survey and the village leaders were asked to update the map, which was then divided into segments, so that each segment included approximately 80 people and one of the segments was chosen at random. All households in the segment were included in the sample sequentially until 80 people were identified. People were eligible for inclusion if they lived in the household at least three months of the year. If the segment did not include 80 people then another segment was chosen at random and sampling continued. If an eligible person was absent, the survey team returned to the household on the same day to examine the individual before leaving the area. If, after repeated visits, the subject could not be examined, information about the believed presence of MSI was collected from relatives or neighbours.

### Assessment of MSI

The survey team visited households door-to-door and conducted the MSI screening in the household. There were three survey teams, each consisting of one physiotherapist, one nurse and one interviewer, and they were assisted in the clusters by a village guide, appointed by the village leaders. The purpose of the study and the examination procedure were explained to the subjects and verbal consent was obtained before examination.

All enumerated participants provided general demographic information and answered a seven-question screening questionnaire about difficulties using their musculoskeletal system, whether they used a mobility aid, whether they had any physical deformity, and how long they had had these symptoms to assess whether they might have an MSI. Participants who answered “yes” to any of the questions were classified as cases, provided that the condition had lasted for more than one month or was considered permanent. This screening tool was developed by orthopaedic surgeons together with physiotherapists and has been shown to have 99% sensitivity and 97% specificity with interobserver Kappa scores of 0.90 for the diagnostic group [13]. Cases were examined and interviewed by the physiotherapist, to observe whether the participant could carry out physical tasks that required use of the musculoskeletal system (i.e. walking, crouching and upper limb motor skills) using a standard protocol. The physiotherapist recorded the diagnosis, area affected, nature of the problem, and aetiology. The severity of the MSI was assessed by the physiotherapists as “mild” if it had little effect on the ability of the area to perform it's normal function, “moderate” if it had a significant effect on the area to perform, but performance was still possible, or “severe” if it had a greater effect on the area to perform and performance was either not possible or almost impossible. The interobserver Kappa score was 0.59 for severity [13].

The physiotherapist also made an assessment of the treatment needed by the case. People with MSI due to trauma were asked whether this trauma occurred during the 1990–1994 war or the episodes that preceded or followed the war.

### Training and pilot study

All team members were Rwandese and spoke the local language (Kinyarwandan). The teams received three weeks of training, including a pilot study. Inter-observer agreement between the physiotherapists was measured before the survey began to ensure that identification of MSIs was of an acceptable standard (i.e. kappa≥0.60).

### Ethics

Ethical approval for this survey was granted by the Independent Ethics Committee in Rwanda and the London School of Hygiene & Tropical Medicine. Permission to proceed was granted by the government, and consent was granted for each cluster visited from the community leader at the province, district, sector and cell level. Informed verbal consent was obtained from the subjects after explanation of the nature and possible consequences of the study. Specific ethical approval was given for verbal consent, due to the high proportion of illiteracy in the population. All people with treatable MSI were referred to a central community rehabilitation centre where clinical members of the study team reviewed and referred the participants for further treatment, as appropriate. The research followed the tenets of the Declaration of Helsinki.

## Results

Out of a total of 8368 people enumerated, 6757 were available for screening and examination (80.8%). Compared to those examined, people not examined were more likely to be male (58% versus 44%) and over 16 years old (62% versus 48%). Since an individual with MSI was allowed up to two separate diagnoses, there were 391 diagnoses in total for 352 cases of MSI giving an overall prevalence of 5.2% (95% CI = 4.5–5.9%). For 374 diagnoses the aetiology was ascertained, and of these, 125 diagnoses (33.4%) in 106 people were of traumatic aetiology.

Out of the 106 people with MSI with a trauma diagnosis, 14 (13.2%) reported the trauma responsible for the MSI occurred during the 1990–1994 war, and 7 (6.6%) reported that the trauma causing their MSI occurred during the violent episodes that preceded or followed the war (Table 1), leaving 85 cases with trauma attributable to other causes. In total, nearly one fifth of trauma-related MSI were attributed to the 1994 war and related violence (19.8%).

**Table 1:** Musculoskeletal impairments caused by genocide-related violence in Rwanda.

Gender	Age at time of survey (2005)	Site of MSI	Nature of MSI	Severity of MSI
**Cases from the 1990–1994 War**				
Male	10	Shoulder	Tendon/muscle/nerve injury	Moderate
Male	51	Hand	Pain following trauma	Moderate
Male	45	Whole body	Pain following trauma	Moderate
Male	47	Hip	Pain following trauma	Moderate
Male	30	Knee	Pain following trauma	Moderate
Male	65	Thigh	Malunited fracture	Moderate
Male	49	Leg	Post traumatic joint stiffness	Severe
Female	41	Hand	Pain following trauma	Moderate
Female	43	Hand	Nerve injury	Moderate
Female	65	Spine	Pain following trauma	Moderate
Female	33	Hand	Lack of arm function following head injury	Moderate
Female	18	Leg	Pain following trauma	Moderate
Female	35	Leg	Pain following trauma	Not specified
Female	18	Thigh	Tendon/muscle/nerve injury	Mild
**Cases from War other than 1990–1994 War**				
Male	50	Lumbar spine	Pain following trauma	Mild
Male	32	Leg	Amputation	Moderate
Male	27	Knee	Pain following trauma	Severe
Male	36	Hip	Pain following trauma	Moderate
Male	32	Fingers	Amputation	Mild
Female	25	Fingers	Amputation	Mild
Female	33	Shoulder	Tendon/muscle/nerve injury	Mild

Of the 14 people with MSI injured during the 1990–1994 war, 7 were male and 7 female (Table 1) while 5 were male and 2 were female among the 7 people with MSI related to the violence preceding and following the war. At the time of their injuries sustained in the 1990–1994 war (assuming most of these were sustained in 1994), one person was an infant and two others were less than 10 years old, nine were adults aged 16–50, and two were aged 54, giving an average age at the time of impairment of 28. Overall, the nature of more than half of the 21 cases was persistent pain following the trauma (52.4%), the traumas affected the lower and upper body and most of the cases were moderate or severe (57.1%).

There was no difference in the age distribution of people with MSI attributable to the 1990–1994 war and related violence compared to all other trauma cases (n = 85), and the sex distribution was also similar (Chi square p-value = 0.73) (Table 2). Cases with MSI attributable to the 1990–1994 war and related violence were most likely to be in the age groups 21–40 years, and this was particularly noticeable for women (Table 2). The proportion of trauma due to the war was lowest in those aged above 60.

**Table 2:** 1990–1994 war-related trauma by age group and gender.

	Males		Females		Total	
Age (years)	1990–1994 war-related trauma (% of trauma)	All other trauma (% of total trauma)	1990–1994 war-related trauma (% of trauma)	All other trauma (% of total trauma)	1990–1994 war-related trauma (% of trauma)	All other trauma (% of total trauma)
0–20	1 (8%)	7 (16%)	2 (22%)	8 (20%)	3 (14%)	15 (18%)
21–40	5 (42%)	17 (38%)	4 (44%)	5 (13%)	9 (43%)	22 (26%)
41–60	5 (42%)	15 (33%)	2 (22%)	14 (35%)	7 (33%)	29 (34%)
61+	1 (8%)	6 (13%)	1 (11%)	13 (33%)	2 (10%)	19 (22%)
Total	12	45	9	40	21	85
Chi square p-value	0.86		0.13		0.37	

The overall prevalence of MSI caused by the 1990–1994 war (0.2%, 95% CI = 0.04–0.3%) and related violence (0.1%, 95% CI = 0.02–0.2%) was 0.3% (95% CI = 0.2–0.4%) in the total population and it was responsible for 5.3% of all cases of MSI. Extrapolating these estimates to the population of Rwanda (n = 8,441,000), there are approximately 16,900 (95% CI = 3,400–25,300) people who have an MSI attributable to the 1990–1994 war and 8,400 (95% CI = 1,700–16,900) who have an MSI as a result of related violence, so that in total 25,300 cases (95% CI = 16,900–33,800) were attributable to these events.

## Discussion

Over a decade after the genocide of 1994, approximately one in twenty people in Rwanda had an MSI, and one in twenty of those cases appeared to be the result of the 1990–1994 war or related violence. Considering the wide-ranging and catastrophic nature of the genocide this may be a surprisingly low proportion and there are several possible explanations. Few people may have sustained lasting injuries, perhaps because they were killed outright, or were unlikely to survive the following decade. Alternatively, people may have recovered from their injuries, since many people reported they had experienced violence during the genocide and its aftermath; One survey of adolescents conducted in 1995 found that 11% reporting having experienced physical injury,[6] while another survey of adults conducted in 2002 found that 18% of respondents reported experiencing physical violence.[1] Other surviving victims of the war may have fled the country and so were not included in the survey sample. Another possibility is that people were reluctant to cite the war or related violence as the cause of their MSI or were not aware that this was the cause of their MSI. We tried to overcome this barrier by ensuring that the examining teams, including the physiotherapists, were Rwandese and people were interviewed in their homes, in an effort to encourage honesty and openness. We also did not use the term genocide in the questionnaire, but instead referred to the “1990–1994 war”.

Comparison of prevalence of disability with other places that have experienced humanitarian emergencies is difficult as estimates of disability are rarely collected, with attention focused more often on the impact on mortality rates.[7], [8], [9], [10], [11] Furthermore, the emergencies vary substantially in length and nature and so generalisation on the impact on disability is not possible. For instance, surveillance in Afghanistan between 2002–2006 showed that there was a high burden of injuries in adults and children due to landmines and unexploded ordnance, resulting in high levels of limb amputation, [12] and a study from Chechnya reported similar results.[13] However, these devices did not feature prominently during the Rwandan genocide. Studies conducted in Rwanda[1], [6] or other post-conflict settings [14], [15] have found a high prevalence of post traumatic stress disorder (PTSD), but have not focussed on MSI. Although PTSD can result in somatic complaints, [16] case eligibility in our study was assessed objectively through a screening questionnaire and physiotherapist though pain was recorded through self-report.

The relatively low prevalence of MSI related to the war observed in this national survey still translates to more than 25,300 directly affected people. The Ministry of Health commissioned this survey to be undertaken so that they can plan services for Rwanda. Many post conflict injuries inflicted by machetes need reconstructive surgery and physiotherapy, while amputations and other major injuries need appliances and rehabilitation services. Many of the cases identified in this survey could therefore have their condition alleviated through appropriate health care, and all cases identified in the study were referred to appropriate services. We cannot compare these estimates to other reports, since no similar survey has been undertaken in Rwanda, and since there has been a delay between conduct of the survey and publication the results may not reflect exactly the situation in Rwanda today. However, the survey was nationally representative and used established sampling techniques, and the examinations and diagnoses were undertaken by trained physiotherapists. The overall prevalence and causes of MSI estimated in the survey were in line with expectations based on other studies. Given that a relatively low proportion of MSI in Rwanda is attributable to the genocide, this means that it may be possible to generalise the prevalence and causes of MSI in Rwanda to other similar African settings for which data are lacking.

In conclusion, the prevalence of MSI related to the 1994 war was lower than anticipated. This finding should not undermine the catastrophic nature of these events, nor the psychological distress that still exists as the result of suffering or witnessing attacks.[1]
